# No evidence from euglycaemic–hyperinsulinaemic clamp studies for greater insulin sensitivity in adults with type 1 diabetes using insulin pump versus multiple daily insulin injections—Post hoc meta‐analysis

**DOI:** 10.1111/dom.16487

**Published:** 2025-07-01

**Authors:** Andrzej S. Januszewski, Jennifer R. Snaith, Agata Grzelka‐Wozniak, Johan R. A. Simonsen, Nirupa Sachithanandan, Glenn M. Ward, David N. O'Neal, Daniel Gordin, Lena M. Thorn, Per‐Henrik Groop, Aleksandra A. Uruska, Dorota A. Zozulinska‐Ziolkiewicz, Alicia J. Jenkins, Jerry R. Greenfield

**Affiliations:** ^1^ Sydney Pharmacy School The University of Sydney Sydney Australia; ^2^ Department of Medicine University of Melbourne Melbourne Australia; ^3^ Clinical Diabetes, Appetite and Metabolism Laboratory Garvan Institute of Medical Research Sydney Australia; ^4^ St Vincent's Clinical Campus University of New South Wales Sydney Australia; ^5^ Department of Diabetes and Endocrinology St Vincent's Hospital Sydney Australia; ^6^ Department of Int. Medicine and Diabetology Poznan University of Medical Sciences Poznan Poland; ^7^ Folkhälsan Research Center Helsinki Finland; ^8^ Research Program for Clinical and Molecular Metabolism University of Helsinki Helsinki Finland; ^9^ Department of Nephrology University of Helsinki and Helsinki University Hospital Helsinki Finland; ^10^ Minerva Foundation Institute for Medical Research Helsinki Finland; ^11^ Joslin Diabetes Center, Harvard Medical School Boston Massachusetts USA; ^12^ Helsinki Hypertension of Excellence University of Helsinki and Helsinki University Hospital Helsinki Finland; ^13^ Department of General Practice and Primary Health Care University of Helsinki Helsinki Finland; ^14^ Diabetes and Vascular Medicine Laboratory Baker Heart and Diabetes Institute Melbourne Australia

**Keywords:** CSII, database research, glycaemic control, insulin resistance, meta‐analysis

## 1 INTRODUCTION

There is evidence suggesting that people with type 1 diabetes using continuous subcutaneous insulin infusion (CSII, or insulin pump therapy) are less insulin resistant than individuals using multiple daily injections (MDI). Indirect evidence for this hypothesis comes from the observed reduction in total daily insulin dose (TDI) in individuals who transition from MDI to CSII.^1^, ^2^ This reduction has been attributed to improved insulin absorption,^3^, ^4^ lower basal insulin requirements^2^ and the avoidance of insulin ‘stacking’, which reduces the risk of hypoglycaemia.^5^

Increased insulin sensitivity (IS) with CSII has also been attributed to steady basal insulin delivery^6^, ^7^ and the administration of smaller, more frequent bolus doses.^8^, ^9^ Additionally, the flexibility of CSII in adjusting basal and bolus doses contributes to better glycaemic control.^10^, ^11^

However, there is a very limited amount of data directly comparing IS in individuals with T1D treated with CSII versus MDI using the ‘gold standard’ euglycaemic–hyperinsulinaemic clamp.^12^

The aim of this study was to compare IS, measured as glucose disposal rate (GDR), and other clinical characteristics—including glycaemic control (HbA1c), body composition (BMI) and lipid profile—in a cross‐sectional analysis of people with T1D managed with CSII or MDI therapy, using euglycaemic–hyperinsulinaemic clamp studies.

## 2 METHODS

We carried out an aggregated (two‐stage) meta‐analysis of glucose disposal rate (GDR) data obtained from 190 unpaired euglycaemic–hyperinsulinaemic clamps conducted between 2006 and 2023 in people with type 1 diabetes (*n* = 38 CSII) in four centres (two in Europe (Finland, Poland) and two in Australia).

### 2.1 Euglycaemic–hyperinsulinaemic clamp

Clamps were conducted according to a standardized protocol,^12^ with minor site‐specific variations. In all centres, intravenous insulin infusion using rapid‐acting analogues (Actrapid in Australia and Poland; NovoRapid in Finland) was administered at a rate of 40 or 60 mU/m^2^/min. Euglycaemia was maintained at 5.0–5.5 mmol/L using 20% (Finland, Poland) or 25% glucose (Australia), with glucose infusion rates adjusted based on plasma glucose monitoring every 5 min using the glucose oxidase method. IS was determined from GDR measured during the final 30 min of the steady‐state period in all studies.

### 2.2 Meta‐analysis

Data were analysed using R (version 4.4.2) with the *metafor* package.^13^ To test the effect of CSII compared with MDI use on IS, a summary mean unpaired difference over all clamp studies was computed, which was the average of the differences at each of the sites, weighted by the inverse of the respective variances. The 95% CI of this summary mean was used to determine whether the difference in mean outcome was of statistical significance (*p* < 0.05).

Heterogeneity was assessed using Cochran's *Q* test statistics and expressed as *I* ^2^ value, which reflects the proportion of variability in effect estimate that is due to heterogeneity rather than chance.

Due to ethical and data governance constraints, individual‐level data could not be shared across jurisdictions. Therefore, we used a two‐stage random‐effects meta‐analysis approach, where multivariable analyses were performed separately at each site, and summary‐level data were then pooled. This allowed for centre‐specific adjustments for covariates while maintaining compliance with local privacy requirements.

## 3 RESULTS

**FIGURE 1:**
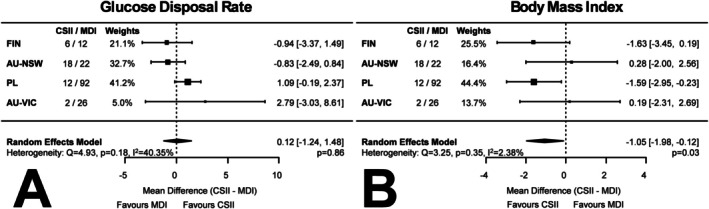
Meta‐analysis comparing (A) glucose disposal rates (GDR) and (B) Body mass index (BMI) in people with type 1 diabetes (*n* = 190) currently receiving continuous subcutaneous insulin infusion (CSII) vs. people with type 1 diabetes on multiple daily injections (MDI). AU‐NSW, Australia–Sydney; AU‐VIC, Australia–Melbourne; FIN, Finland‐Helsinki; PL, Poland‐Poznan.

General characteristics of participants and meta‐analysis results are shown in Table 1. There was no difference in HbA1c between CSII and MDI users, although with substantial heterogeneity (−0.51 [−1.43, 0.41], *p* = 0.28, *I* ^2^ = 81.91%, *p* = 0.0003). Body Mass Index (BMI) was lower in individuals using CSII vs. MDI (−1.05 [−1.98, −0.12] kg/m^2^, *p* = 0.03, *I* ^2^ = 2.38%, *p* = 0.35) (Figure 1B). None of the other parameters (demographics, clinical characteristic) were different in meta‐analysis between CSII and MDI users (Table 1).

**TABLE 1:** Characteristics of individuals with type 1 diabetes (T1D) undergoing clamp procedure in four centres and CSII vs. MDI meta‐analysis results.

	Finland (Helsinki) 2021–2023	Australia‐VIC (Melbourne) 2006–2007	Poland (Poznań) 2013–2015	Australia‐NSW (Sydney) 2020–2021	RF model	*p*‐value*	*I* ^2^ (%)	*p*‐value**
CSII/MDI	6/12	2/26	12/92	18/22	‐	**<0.0001**	‐	‐
F/M	8/10	13/15	32/72	16/24	‐	0.23	‐	‐
Age (years)	51 ± 11	40 ± 13	34 ± 7	37 ± 8	−2.16 (−8.17, 3.85)	0.48	61.76%	**0.049**
Diabetes duration (years)	37 ± 12	21 ± 11	10 ± 4	23 ± 9	1.50 (−2.02, 5.03)	0.40	46.46%	0.13
TDI (IU/kg)	0.54 ± 0.18	0.53 ± 0.24	0.50 ± 0.19	0.63 ± 0.20	0.01 (−0.09, 0.12)	0.82	61.59%	0.056
HbA1c (%)	7.1 ± 0.9	7.7 ± 1.4	7.7 ± 1.5	7.5 ± 0.9	−0.51 (−1.43, 0.41)	0.28	81.91%	**0.0003**
BMI (kg/m^2^)	26.6 ± 2.0	25.2 ± 3.3	24.9 ± 3.6	26.4 ± 3.8	−1.05 (−1.98, −0.12)	**0.03**	2.38%	0.35
WHR	0.90 ± 0.11	0.86 ± 0.10	0.86 ± 0.08	0.89 ± 0.08	−0.02 (−0.07, 0.04)	0.55	69.88%	**0.013**
SBP (mmHg)	143 ± 21	125 ± 13	123 ± 14	119 ± 12	−2.31 (−11,29, 6.67)	0.61	72.29%	**0.011**
DBP (mmHg)	83 ± 12	71 ± 9	78 ± 10	73 ± 8	−1.67 (−6.69, 3.35)	0.51	68.93%	**0.016**
TC (mmol/L)	3.9 ± 1.2	4.8 ± 1.4	4.9 ± 1.0	4.3 ± 0.8	−0.15 (−0.46, 0.16)	0.34	0%	0.95
TG (mmol/L)	1.3 (0.8–1.5)	0.9 (0.7–1.2)	0.9 (0.6–1.3)	0.8 (0.6–1.0)	−0.07 (−0.37, 0.23)	0.65	50.10%	0.12
LDL‐C (mmol/L)	2.2 ± 1.1	2.9 ± 1.2	2.9 ± 0.8	2.6 ± 0.6	−0.17 (−0.44, 0.11)	0.24	0%	0.47
HDL‐C (mmol/L)	1.4 ± 0.5	1.5 ± 0.3	1.7 ± 0.4	1.3 ± 0.4	0.06 (−0.22, 0.33)	0.69	75.26%	**0.006**
eGFR (CKD‐EPI 2021)	75 (62–104)	88 (81–101)	112 (99–118)	106 (91–112)	2.26 (−4.31, 8.82)	0.50	0%	0.92
Smoking (current, *n* / %)	1 / 5.6	7 / 25.0	33 / 31.7	0 / 0.0	‐	**<0.0001**	‐	‐
Diabetes complications (*n* / %)	12 / 66.7	10 / 35.7	36 / 34.6	17 / 42.5	‐	0.07	‐	‐

*Note*: Diabetes complications were defined as either micro‐ or macrovascular. Bold value indicates *p* < 0.5

Abbreviations: BMI, body mass index; CKD‐EPI, Chronic Kidney Disease Epidemiology Collaboration; CSII, continuous subcutaneous insulin infusion; eGFR, estimated glomerular filtration rate; LDL‐C/HDL‐C, low/high‐density lipoprotein cholesterol; MDI, multiple daily injections; RF, random‐effects CSII versus MDI; SBP/DBP, systolic/diastolic blood pressure; TC, total cholesterol; TDI, total daily insulin dose; TG, triglycerides; WHR, waist‐to‐hip ratio.

* Meta‐analysis or *χ* ^2^ *p*‐value.

** Heterogeneity *p*‐value.

There was no difference in GDR between individuals treated with CSII versus MDI (random‐effects model 0.12 [−1.24, 1.48] mg/kg/min, *p* = 0.86, *I* ^2^ = 40.35%, *p* = 0.18). (Figure 1A). *Z*‐score standardized GDR results were similar (data not shown). To account for potential confounding, we conducted adjusted analyses using linear model residuals that included T1D duration, age, total daily insulin dose, blood pressure, lipid parameters and eGFR; these adjustments did not materially alter the results (Table S1), suggesting no significant difference in IS between CSII and MDI users across a range of sensitivity‐related metrics.

## 4 DISCUSSION

In this cross‐sectional study of 190 individuals with type 1 diabetes undergoing ‘gold standard’ euglycemic–hyperinsulinemic clamp studies, IS, as reflected by GDR, did not differ significantly between CSII and MDI users. There is a limited body of evidence focused on the objective measurement of IS in people with type 1 diabetes treated with CSII or MDI. In two studies published in 1984 and 1985, IS was reported to be higher compared to the preceding period on MDI, following the CSII commencement.^14^, ^15^ These studies, with a combined sample of 18 subjects, used paired comparisons and reflected the technology and insulin formulations of their time. We can only speculate that advancements in insulin manufacturing, pump technology and glucose administration algorithms over the past decades might influence the outcomes if similar studies were conducted today.

BMI was significantly lower in CSII users compared to those treated with MDI. This finding suggests that the choice between CSII and MDI in adults with type 1 diabetes should prioritize other factors, such as lifestyle compatibility, glycaemic variability or patient preference, rather than changes in insulin sensitivity.

Although there is some indirect evidence suggesting better IS in CSII users, the lack of significant GDR differences in our study is not entirely surprising. Both CSII and MDI deliver insulin to the same extracellular space, bypassing the hepatic circulation and causing peripheral hyperinsulinaemia. This hyperinsulinaemia is a primary driver of insulin resistance in type 1 diabetes.^16^ We did not observe a significant difference in TDI nor HbA1c favouring CSII over MDI. While reductions in TDI have been observed shortly after transitioning from MDI to CSII,^14^, ^15^ our study included individuals already established on their respective insulin regimens, and we did not have access to longitudinal data capturing pre‐ and post‐transition insulin doses. This limits our ability to assess whether initial reductions in TDI with CSII are sustained over time. We also evaluated standardized GDR scores and conducted multivariable‐adjusted analyses incorporating clinical covariates associated with insulin sensitivity. These additional analyses supported our primary finding of no significant difference in IS between CSII and MDI users.

CSII use may contribute to lower BMI. Considering CSII as part of a broader metabolic health strategy could help reduce cardiovascular risks associated with higher BMI in type 1 diabetes patients. Over time, lower BMI may lead to reduced insulin resistance, improved blood pressure regulation and more favourable lipids, reinforcing the broader health benefits of CSII. Indeed, CSII use has been associated with significantly lower chronic complication rates in people with T1D.^17^, ^18^, ^19^

### 4.1 Study strengths and limitations

This study is the result of a multicentre international collaboration, involving a large number of type 1 diabetes patients on CSII and MDI undergoing clamp procedures. The clamp protocol was consistent across all sites, which strengthens the validity of the findings. However, we acknowledge several limitations. As the original clamp studies used in this meta‐analysis were not designed to compare IS in people with type 1 diabetes on MDI vs. CSII, the present findings must be considered as *post hoc* analyses. We also lacked data on the length of the participant's present insulin therapy mode and physical exercise,^20^ which both might affect IS, as well as data on IS prior to the initiation of CSII therapy. This is a cross‐sectional study with a limited number of people with type 1 diabetes on CSII; hence, heterogeneity in various parameters may have influenced the results. Additionally, during the period when data were collected (2006–2023), insulin pumps underwent substantial technological and software advancements. Ongoing improvements in pump technology, such as the integration of closed‐loop systems and the use of adjunct glucose control therapies, may offer benefits not captured in the current analysis. We have also clarified the clamp protocol details in the Methods section, including insulin type and infusion rates, glucose targets and measurement methods, and glucose infusion procedures, to support transparency and reproducibility. It is thus crucial to understand potential mechanisms behind improved insulin treatment regimen that new technology may provide.

A limitation of our approach was that individual‐level data could not be transferred between centres due to ethical and privacy regulations. Consequently, direct comparisons of baseline characteristics across centres were not feasible. Nevertheless, site‐specific adjustments and the random‐effects model helped to account for heterogeneity, partially mitigating this limitation. Despite prior suggestions, we could not show an ameliorated insulin sensitivity between insulin pump and MDI treatments, using 'gold standard' methodology.

We recognize that some readers may expect a pooled multivariable regression analysis; however, due to the inability to share individual‐level data across jurisdictions, this was not feasible. To address this, we conducted within‐centre linear models to adjust GDR for potential confounders (including age, diabetes duration, TDI, eGFR, lipid profile and blood pressure), followed by meta‐analysis of the adjusted results. This allowed us to assess the robustness of our findings despite the unpaired design and site‐level analysis. Future longitudinal studies are merited to compare IS between MDI and CSII, particularly those that assess both central (hepatic) and peripheral (muscle) IS. Such studies could provide a more comprehensive understanding of the metabolic benefits of these insulin delivery modalities.

## AUTHOR CONTRIBUTIONS

**ASJ:** Design, conduct, data collection, analysis, writing. **JRS:** Conduct, data collection, writing. **AGW:** Conduct, data collection, writing. **JRAS:** Conduct, data collection, analysis, writing. **NS:** conduct, data collection, writing. **GMW:** Conduct, data collection, writing. **DNO:** Conduct, data collection, writing. **DG:** Conduct, data collection, writing. **LMT:** Conduct, data collection, writing. **PHG:** Design, conduct, data collection, writing. **AAU:** Design, conduct, data collection, analysis, writing. **DAZZ:** Design, conduct, data collection, analysis, writing. **AJJ:** Design, writing. **JRG:** Design, conduct, data collection, analysis, writing.

## PEER REVIEW

The peer review history for this article is available at https://www.webofscience.com/api/gateway/wos/peer‐review/10.1111/dom.16487.

## Supporting information


**Table S1.** Meta‐analysis comparing GDR adjusted for various confounders. RF—random‐effects CSII versus MDI, *—meta‐analysis p‐value, **—heterogeneity p‐value. Abbreviations: CSII—continuous subcutaneous insulin infusion, MDI—multiple daily injections, TDI—total daily insulin dose, BMI—body mass index, WHR—waist‐to‐hip ratio, SBP/DBP—systolic/diastolic blood pressure, TC—total cholesterol, TG—triglycerides, LDL‐C/HDL‐C—low/high‐density lipoprotein cholesterol, eGFR—estimated glomerular filtration rate, CKD‐EPI—Chronic Kidney Disease Epidemiology Collaboration. Diabetes complications were defined as either micro‐ or macrovascular.

## Data Availability

The datasets analysed in this study are not publicly available due to ethical and data governance restrictions imposed by the participating institutions, which limit individual‐level data sharing across jurisdictions.

## References

[1] Nicolajsen T , Samuelsson A , Hanas R . Insulin doses before and one year after pump start: children have a reversed dawn phenomenon. J Diabetes Sci Technol. 2012;6(3):589‐594. 22768890 10.1177/193229681200600314 PMC3440064

[2] Paldus B , Lee MH , O'Neal DN . Insulin pumps in general practice. Aust Prescr. 2018;41(6):186‐190. 30670886 10.18773/austprescr.2018.056 PMC6299172

[3] Hauzenberger JR , Hipszer BR , Loeum C , et al. Detailed analysis of insulin absorption variability and the tissue response to continuous subcutaneous insulin infusion catheter implantation in swine. Diabetes Technol Ther. 2017;19(11):641‐650. 28981324 10.1089/dia.2017.0175 PMC5689134

[4] Liu D , Moberg E , Wredling R , Lins PE , Adamson U . Insulin absorption is faster when keeping the infusion site in use for three days during continuous subcutaneous insulin infusion. Diabetes Res Clin Pract. 1991;12(1):19‐24. 1855437 10.1016/0168-8227(91)90126-x

[5] Walsh J , Roberts R , Heinemann L . Confusion regarding duration of insulin action: a potential source for major insulin dose errors by bolus calculators. J Diabetes Sci Technol. 2014;8(1):170‐178. 24876553 10.1177/1932296813514319 PMC4454113

[6] Bergenstal RM , Tamborlane WV , Ahmann A , et al. Effectiveness of sensor‐augmented insulin‐pump therapy in type 1 diabetes. N Engl J Med. 2010;363(4):311‐320. 20587585 10.1056/NEJMoa1002853

[7] Pickup JC , Sutton AJ . Severe hypoglycaemia and glycaemic control in type 1 diabetes: meta‐analysis of multiple daily insulin injections compared with continuous subcutaneous insulin infusion. Diabet Med. 2008;25(7):765‐774. 18644063 10.1111/j.1464-5491.2008.02486.x

[8] Pickup JC . Insulin‐pump therapy for type 1 diabetes mellitus. N Engl J Med. 2012;366(17):1616‐1624. 22533577 10.1056/NEJMct1113948

[9] Weissberg‐Benchell J , Antisdel‐Lomaglio J , Seshadri R . Insulin pump therapy: a meta‐analysis. Diabetes Care. 2003;26(4):1079‐1087. 12663577 10.2337/diacare.26.4.1079

[10] Cummins E , Royle P , Snaith A , et al. Clinical effectiveness and cost‐effectiveness of continuous subcutaneous insulin infusion for diabetes: systematic review and economic evaluation. Health Technol Assess. 2010;14(11). iii‐iv, xi‐xvi:1‐181. 10.3310/hta14110 20223123

[11] Misso ML , Egberts KJ , Page M , et al. Continuous subcutaneous insulin infusion (CSII) versus multiple insulin injections for type 1 diabetes mellitus. Cochrane Database Syst Rev. 2010;(1):CD005103. 20091571 10.1002/14651858.CD005103.pub2 PMC12582037

[12] DeFronzo RA , Tobin JD , Andres R . Glucose clamp technique: a method for quantifying insulin secretion and resistance. Am J Physiol. 1979;237(3):E214‐E223. 382871 10.1152/ajpendo.1979.237.3.E214

[13] Viechtbauer W . Conducting meta‐analyses in R with the metafor package. J Stat Softw. 2010;36(3):1‐48.

[14] Simonson DC , Tamborlane WV , Sherwin RS , et al. Improved insulin sensitivity in patients with type I diabetes mellitus after CSII. Diabetes. 1985;34(Suppl 3):80‐86. doi:10.2337/diab.34.3.S80 3894130

[15] Yki‐Jarvinen H , Koivisto VA . Continuous subcutaneous insulin infusion therapy decreases insulin resistance in type 1 diabetes. J Clin Endocrinol Metab. 1984;58(4):659‐666. 6365945 10.1210/jcem-58-4-659

[16] Gregory JM , Cherrington AD , Moore DJ . The peripheral peril: injected insulin induces insulin insensitivity in type 1 diabetes. Diabetes. 2020;69(5):837‐847. 32312900 10.2337/dbi19-0026 PMC7171956

[17] Steineck I , Cederholm J , Eliasson B , et al. Insulin pump therapy, multiple daily injections, and cardiovascular mortality in 18,168 people with type 1 diabetes: observational study. BMJ. 2015;350:h3234. 26100640 10.1136/bmj.h3234 PMC4476263

[18] Virk SA , Donaghue KC , Wong TY , Craig ME . Interventions for diabetic retinopathy in type 1 diabetes: systematic review and meta‐analysis. Am J Ophthalmol. 2015;160(5):1055‐1064e4. 26210869 10.1016/j.ajo.2015.07.024

[19] Zabeen B , Craig ME , Virk SA , et al. Insulin pump therapy is associated with lower rates of retinopathy and peripheral nerve abnormality. PLoS One. 2016;11(4):e0153033. 27050468 10.1371/journal.pone.0153033 PMC4822832

[20] Helleputte S , Stautemas J , de Craemer M , et al. Physical activity and sedentary behaviour in relation to body composition, estimated insulin sensitivity and arterial stiffness in adults with type 1 diabetes. Diabetes Res Clin Pract. 2024;217:111860. 39293499 10.1016/j.diabres.2024.111860

