# Supplementary material for: No evidence from euglycaemic–hyperinsulinaemic clamp studies for greater insulin sensitivity in adults with type 1 diabetes using insulin pump versus multiple daily insulin injections—Post hoc meta‐analysis
Source: Diabetes Obes Metab. 2025 Jul 1;27(9):5322–6. doi: 10.1111/dom.16487 (PMC12327168; doi:10.1111/dom.16487)
Supplement: Supplementary file 1 — Table S1. Meta‐analysis comparing GDR adjusted for various confounders. RF—random‐effects CSII versus MDI, *—meta‐analysis p‐value, **—heterogeneity p‐value. Abbreviations: CSII—continuous subcutaneous insulin infusion, MDI—multiple daily injections, TDI—total daily insulin dose, BMI—body mass index, WHR—waist‐to‐hip ratio, SBP/DBP—systolic/diastolic blood pressure, TC—total cholesterol, TG—triglycerides, LDL‐C/HDL‐C—low/high‐density lipoprotein cholesterol, eGFR—estimated glomerular filtration rate, CKD‐EPI—Chronic Kidney Disease Epidemiology Collaboration. Diabetes complications were defined as either micro‐ or macrovascular. [file DOM-27-5322-s001.docx]

**Supplementary Table 1**

Meta-analysis comparing GDR adjusted for various confounders. RF – random effects CSII vs. MDI, * - meta-analysis p-value, ** - heterogeneity p-value. Abbreviations: CSII – continuous subcutaneous insulin infusion, MDI – multiple daily injections, TDI – total daily insulin dose, BMI – body mass index, WHR – waist to hip ratio, SBP/DBP – systolic/diastolic blood pressure, TC – total cholesterol, TG – triglycerides, LDL-C/HDL-C – low/high density lipoprotein cholesterol, eGFR – estimated glomerular filtration rate, CKD-EPI - Chronic Kidney Disease Epidemiology Collaboration. Diabetes complications were defined as either micro- or macrovascular.

| **Model covariates** | **RF model** | **p-value*** | **I^2^ (%)** | **p-value**** |
| --- | --- | --- | --- | --- |
| T1D duration | 0.12 (-1.14 – 1.37) | 0.85 | 31.7 | 0.25 |
| TC | 0.06 (-1.34 – 1.45) | 0.93 | 43.9 | 0.15 |
| TC + T1D duration | 0.07 (-1.20 – 1.33) | 0.92 | 34.7 | 0.23 |
| HDL-C | 0.13 (-1.02 – 1.29) | 0.82 | 28.8 | 0.33 |
| SBP | 0.26 (-0.99 – 1.51) | 0.68 | 32.5 | 0.25 |
| DBP | 0.30 (-0.82 – 1.42) | 0.60 | 23.6 | 0.32 |
| WHR | -0.04 (-1.36 – 1.28) | 0.95 | 41.0 | 0.19 |
| TDI | 0.07 (-1.21 – 1.36) | 0.91 | 41.9 | 0.15 |
| Age + Sex | -0.17 (-1.37, 1.03) | 0.78 | 26.57 | 0.29 |
| Age + Sex + HbA1c | -0.17 (-1.47, 1.12) | 0.79 | 40.11 | 0.20 |
| Age + Sex + HbA1c + smoking + BMI | 0.02 (-0.93, 0.97) | 0.97 | 22.31 | 0.35 |
| Age + Sex + HbA1c + smoking + BMI + CX | -0.07 (-1.09, 0.94) | 0.89 | 36.97 | 0.22 |
| Age + Sex + HbA1c + smoking + BMI + eGFR | -0.03 (-1.02, 0.95) | 0.95 | 29.86 | 0.28 |
| T1D duration + TDI + BMI + SBP | -0.06 (-1.19, 1.06) | 0.91 | 35.02 | 0.20 |
| Age + TDI + BMI + SBP | -0.23 (-1.18, 0.73) | 0.64 | 19.58 | 0.28 |
